# Supplementary figures and images for: An age-stratified serosurvey against purified Salmonella enterica serovar Typhi antigens in the Lao People´s Democratic Republic
Source: PLoS Negl Trop Dis. 2021 Dec 13;15(12):e0010017. doi: 10.1371/journal.pntd.0010017 (PMC8700045; doi:10.1371/journal.pntd.0010017)

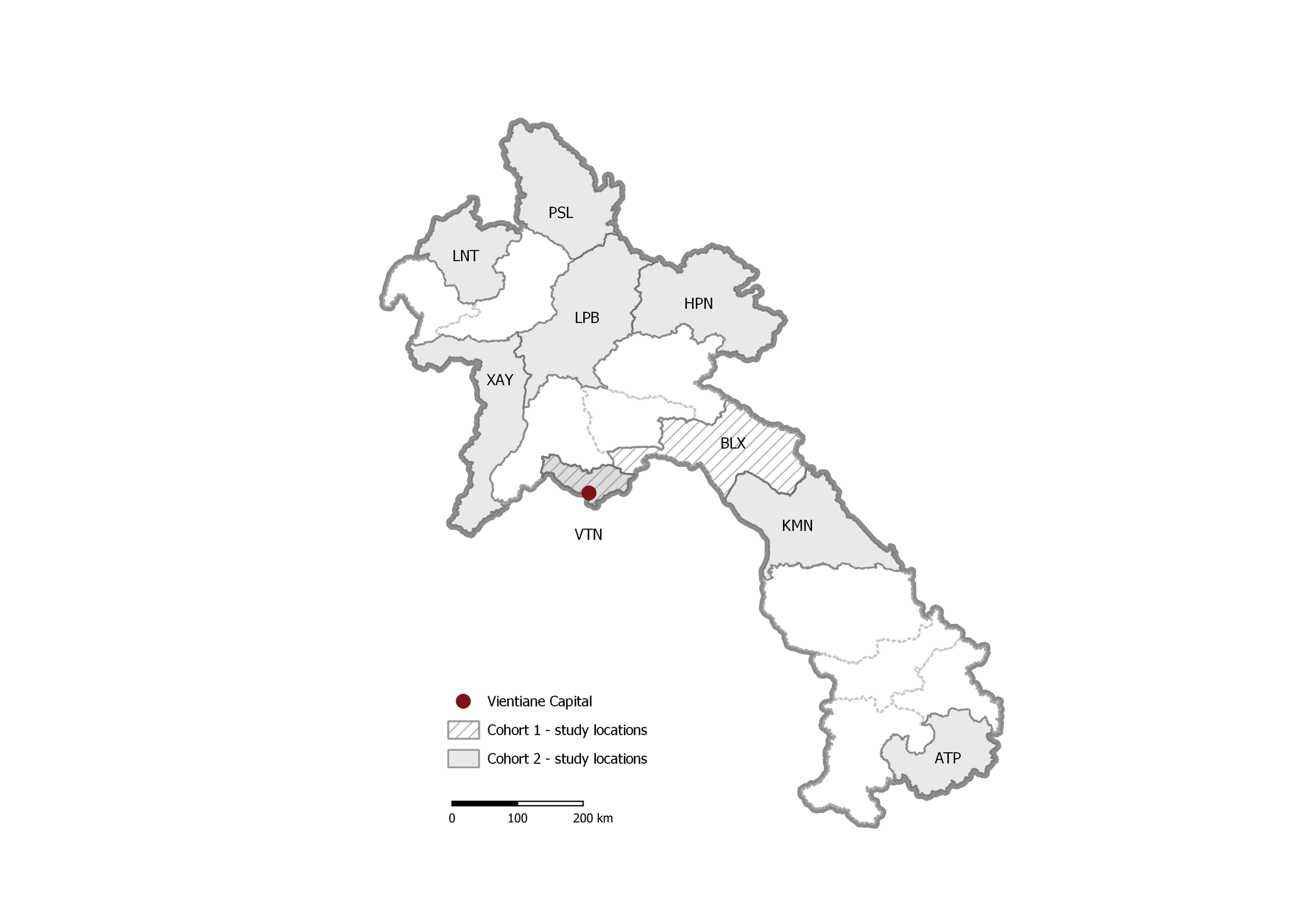

Supplement: S1 Fig — PSL = Phongsaly, LNT = Luang Namtha, HPN = Huaphan, LPB = Luang Prabang, XAY = Xayabouli, VTN = Vientiane, BLX = Bolikhamxay, KHM = Khammouane, ATP = Attapeu. The map was created with QGIS (QGIS Development Team, 2018). The data regarding the administrative boundaries of Lao PDR were obtained from the Humanitarian Data Exchange website https://data.humdata.org/dataset/lao-admin-boundaries, dataset provided by the National Geographic Department of Lao PDR, 2019) and recreated under a CC BY-IGO license. Projection used: EPSG 4326 –WGS 84. (TIF) [file pntd.0010017.s001.tif]

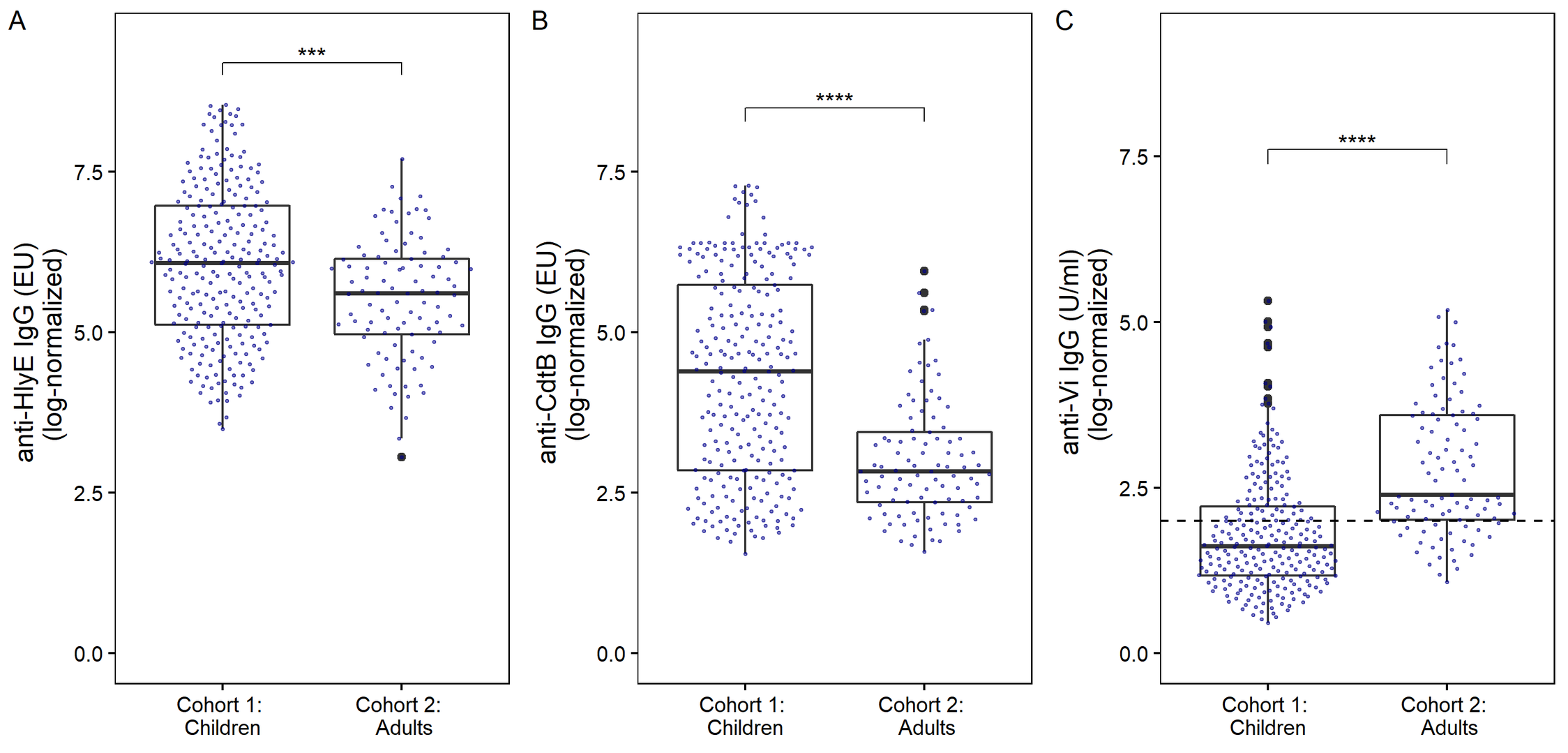

Supplement: S2 Fig — Each dot shows the antibody titer of an individual sample for (A) anti-HlyE IgG, (B) anti-CdtB IgG, and (C) anti-Vi IgG with an underlying boxplot. The dashed line in panel C represents the censoring limit, all data points below were treated as left-censored data. Differences between groups were assessed using Wilcoxon rank sum test followed by Dunn’s post-hoc test with Bonferroni correction: ***p<0.001, ****p<0.0001. (TIF) [file pntd.0010017.s002.tif]

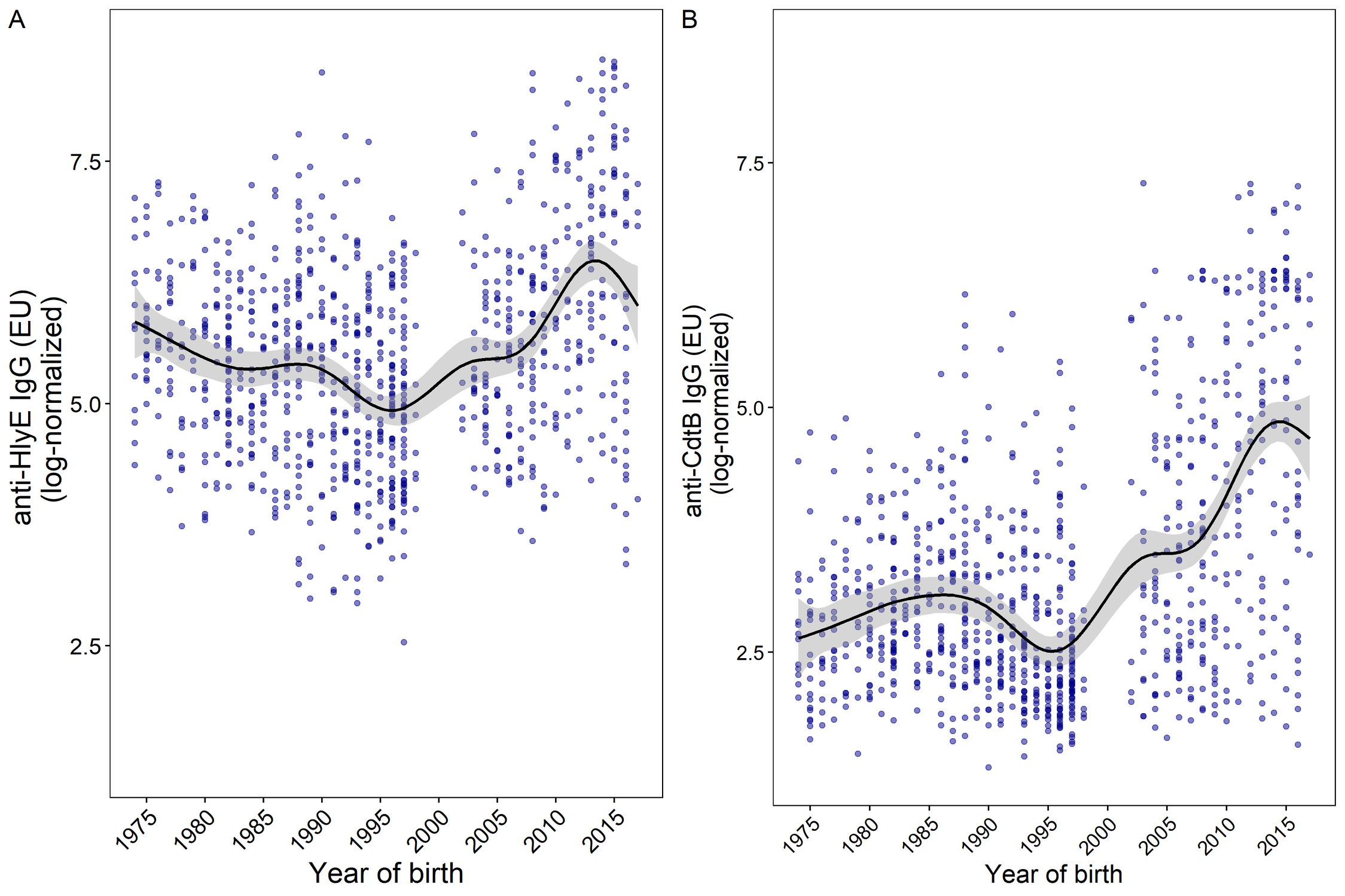

Supplement: S3 Fig — Non-linear smooths were fitted for birth year in the model for anti-HlyE IgG (A) and anti-CdtB IgG (B) data. Shaded bands represent the pointwise 95%-confidence interval. (TIF) [file pntd.0010017.s003.tif]

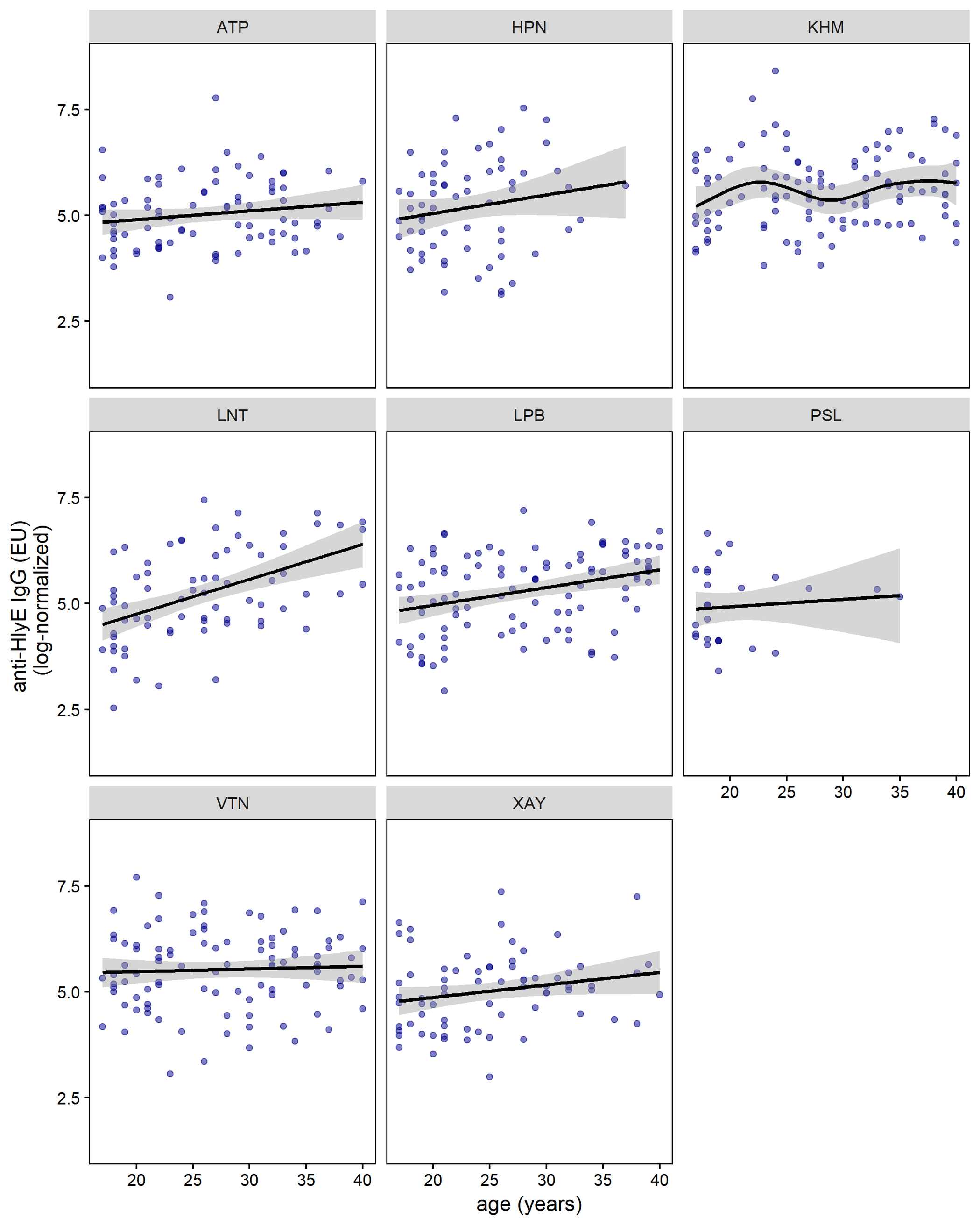

Supplement: S4 Fig — Shaded bands represent the pointwise 95%-confidence interval. ATP = Attapeu, HPN = Huaphan, KHM = Khammouane, LNT = Luang Namtha, LPB = Luang Prabang, VTN = Vientiane, PSL = Phongsaly, XAY = Xayabouli. (TIF) [file pntd.0010017.s004.tif]

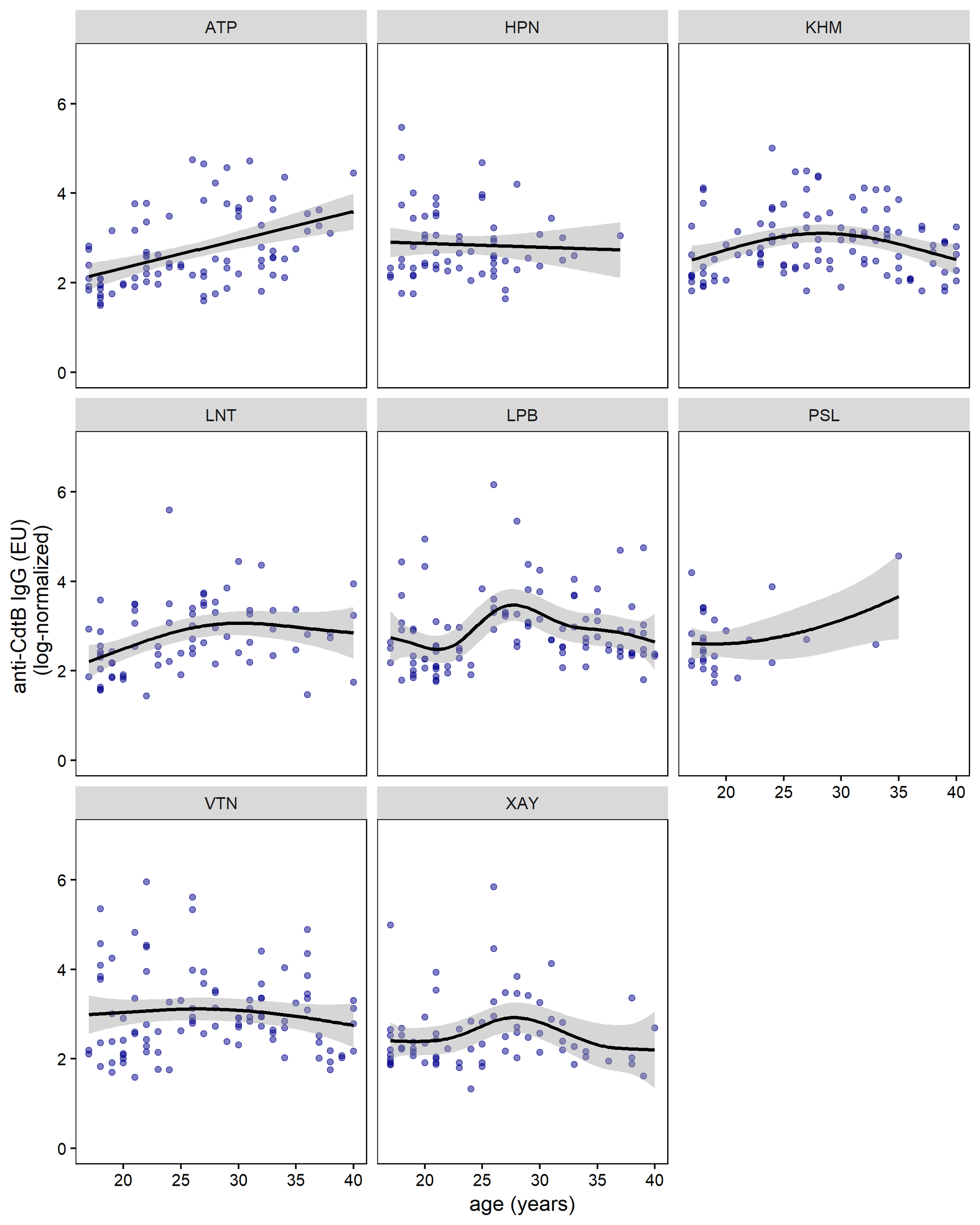

Supplement: S5 Fig — Shaded bands represent the pointwise 95%-confidence interval. ATP = Attapeu, HPN = Huaphan, KHM = Khammouane, LNT = Luang Namtha, LPB = Luang Prabang, VTN = Vientiane, PSL = Phongsaly, XAY = Xayabouli. (TIF) [file pntd.0010017.s005.tif]

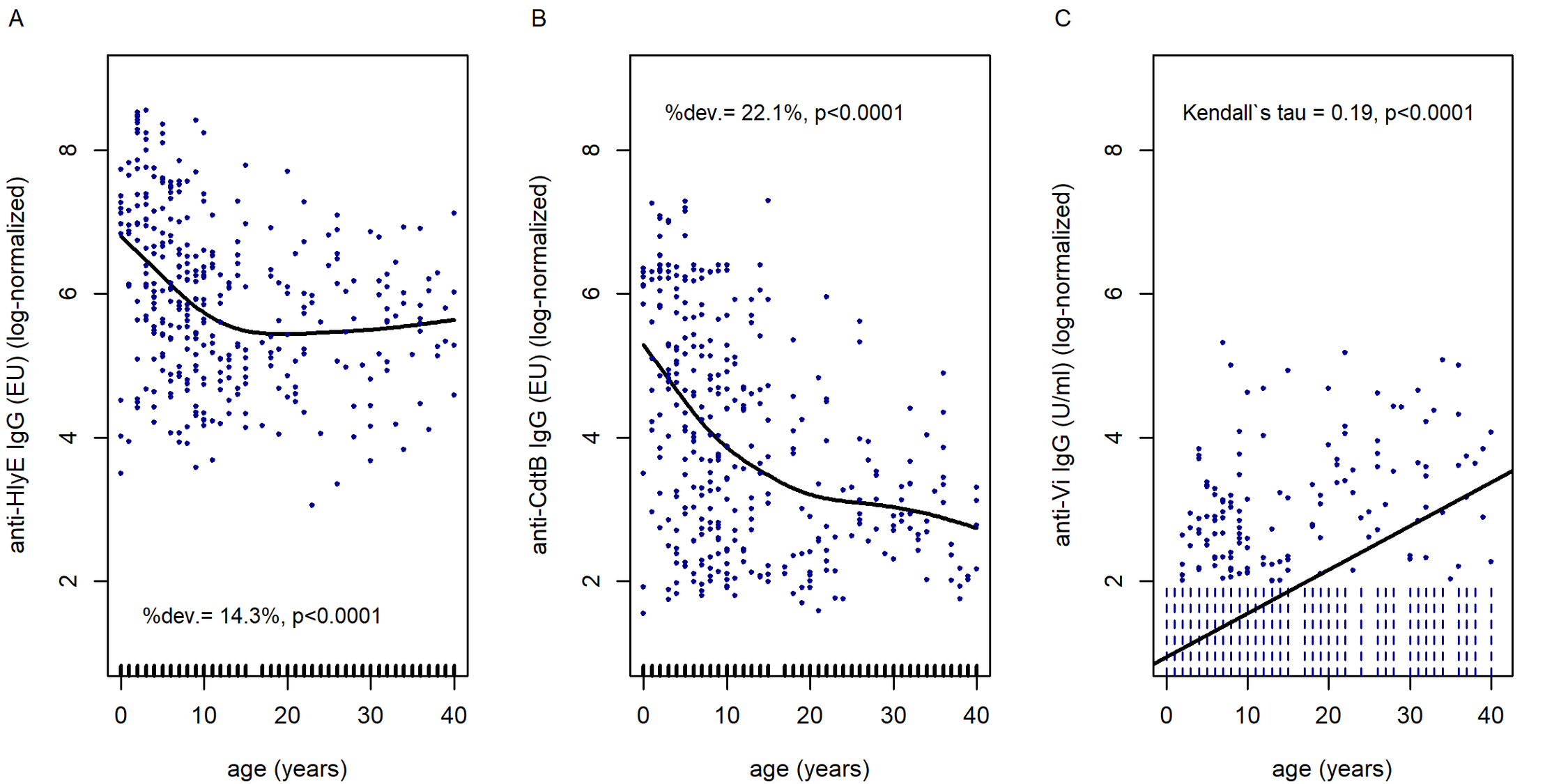

Supplement: S6 Fig — Non-linear smooths were fitted for age in the model for anti-HlyE IgG (A) and anti-CdtB IgG (B) data. The tick marks on the x-axis are observed data points. In panel C, the Akritas-Thiel-Sen regression line relating to the anti-Vi IgG titer data as function of age was plotted in order to account for the censored values (censored observations were plotted as vertical dashed lines). %dev. = the percent of the total model deviance explained. (TIF) [file pntd.0010017.s006.tif]

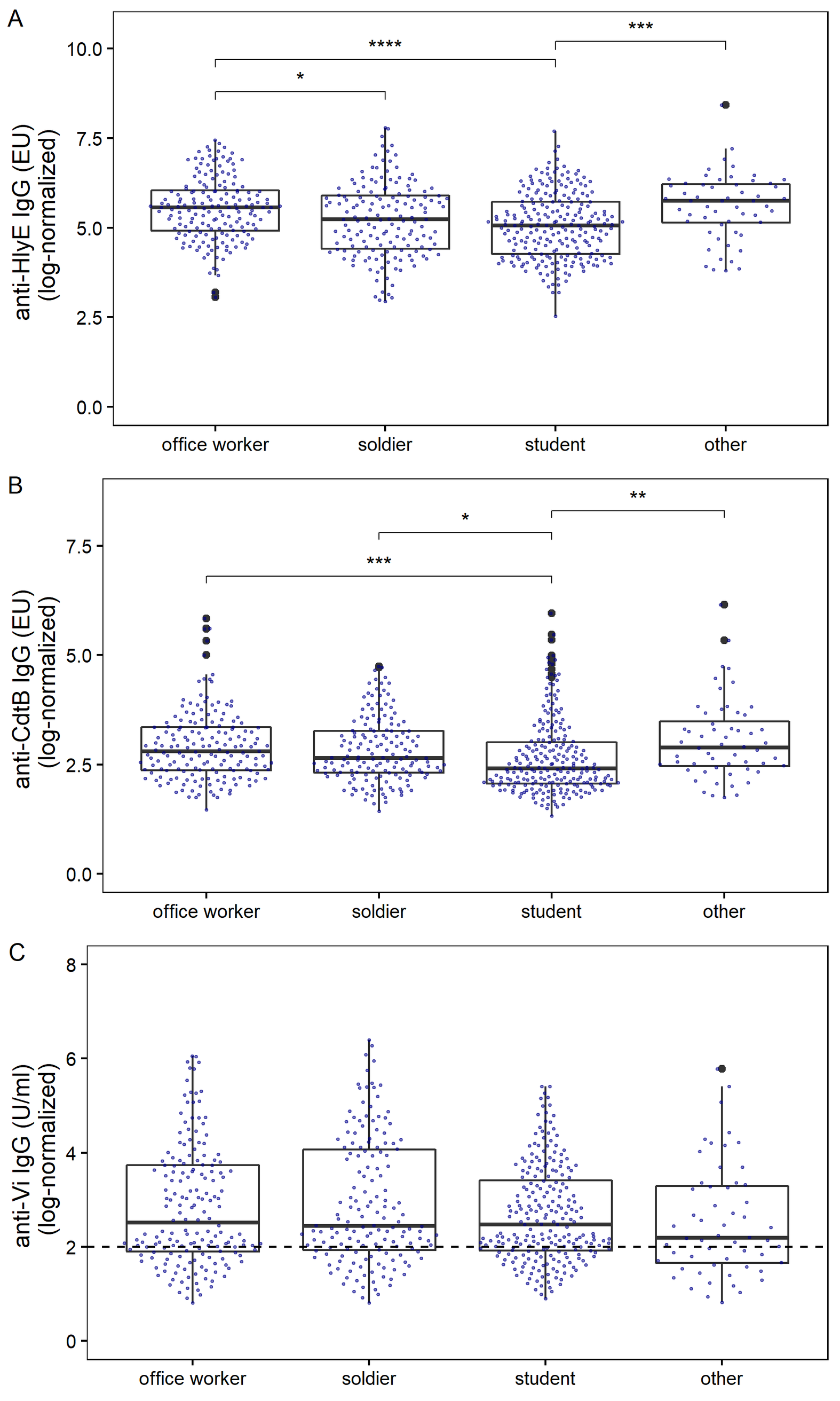

Supplement: S7 Fig — Each dot shows the measurement of an individual sample for (A) anti-HlyE IgG, (B) anti-CdtB IgG and (C) anti-Vi IgG with an underlying boxplot. Differences between groups were assessed using Kruskal-Wallis test followed by Dunn’s post-hoc test with Bonferroni correction: *p<0.05, **p<0.01, ***p<0.001, ****p<0.0001. If not specified otherwise, differences in titer data were non-significant. Participants whose occupation is not specified are grouped into “other”. The dashed line in panel C represents the censoring limit, all data points below were treated as left-censored data. (TIF) [file pntd.0010017.s007.tif]
