# Supplementary material for: An age-stratified serosurvey against purified Salmonella enterica serovar Typhi antigens in the Lao People´s Democratic Republic
Source: PLoS Negl Trop Dis. 2021 Dec 13;15(12):e0010017. doi: 10.1371/journal.pntd.0010017 (PMC8700045; doi:10.1371/journal.pntd.0010017)
Supplement: Attachment — Submitted filename: Cover letter_210720.doc [file pntd.0010017.s008.doc]

Professor Stephen Baker

Sgb47@medschl.cam.ac.uk

21^st^ July 2021

Re: Submission to PLoS neglected tropical diseases

Dear editor,

On behalf of all authors, I wish the midified manuscript entitled **“An age-stratified serosurvey against purified *Salmonella enterica* serovar Typhi antigens in the Lao People´s Democratic Republic”** by Lisa Hefele et al, for which I am the corresponding author, to be considered for publication as a research article in PLoS neglected tropical diseases. We thank the editor and reviewers for their support and have addressed each comment in turn below.

Reviewer #1:

The objectives as set out are noble and would answer key research questions in this area. The study design should have had an element of longitudinal collection of serological data on the study population to understand how long the markers of typhoid exposure persist in the individuals and how this relates to patient presentation or carriage status Sample size and analytical framework are well designed, and no concerns on ethics.

*Given the study limitations we thank the reviewer for their comments.*

Reviewer #2:

The methodology of the study is clearly presented and articulated.

However, the specificity of the 3 antibodies for S. Typhi (vs other non-typhoidal serovars for instance), and their detectability in the serum are not compared or discussed. This absence limits the conclusion of the study but could be included as an additional experiment testing the presence of these antibodies in the blood of patients after typhoid fever was detected (for this purpose, the sample collection could be unrestrained to Lao if necessary). Alternatively, references to previously published work providing this comparison (if existing) should be included and discussed.

*Both the protein antigens HlyE and CdtB were identified in previous research to be able to differentiate between typhoid fever cases and controls (Andrews et al., 2019 and Sharma et al., 2018) (see lines 71 to 73 in our Introduction). Both HlyE and CdtB are expressed in S. Typhi, S. Paratyphi A and rarely in other Salmonella spp. The Vi antigen, the target for previous vaccine development, represents another important immunogen. Currently, the antibody dynamics in relation to typhoid fever disease progression and recovery are being investigated and therefore, the interpretation of the antibody responses is challenging.*

*We agree with the reviewers that these issues should be discussed in our manuscript and added the following text:*

*Lines 71 to 77:*

*“Antibodies against hemolysin E (anti-HlyE) and cytolethal distending toxin subunit B homolog (anti-CdtB) have been identified as potential biomarkers for identifying typhoid fever cases/exposure (ref). HlyE and CdtB are expressed in S. Typhi and S. Paratyphi A, but are rarely found in other Salmonella spp(ref). Additionally, antibody responses against the capsular polysaccharide Vi antigen (anti-Vi), the major component of TCVs, have also been used to assess exposure (ref). The Vi antigen is only present in S. Typhi, S. Dublin and S. Paratyphi C, but is absent from S. Paratyphi A and most of the gastroenteritis-causing serovars(ref).”*

*References include:*

*Charles et al., 2010*

*Characterization of Anti-Salmonella enterica Serotype Typhi Antibody Responses in Bacteremic Bangladeshi Patients by an Immunoaffinity Proteomics-Based Technology*

*Charles et al., 2014;*

*Immunoproteomic Analysis of Antibody in Lymphocyte Supernatant in Patients with Typhoid Fever in Bangladesh*

*Liang et al., 2013*

*Immune profiling with a Salmonella Typhi antigen microarray identifies new diagnostic biomarkers of human typhoid*

*Andrews et al., 2019 ;*

*Plasma Immunoglobulin A Responses Against 2*

*Salmonella Typhi Antigens Identify Patients With*

*Typhoid Fever*

*Sharma et al., 2018*

*Serodiagnostic evaluation of recombinant CdtB of S. Typhi as a potential candidate for acute typhoid*

*Lines 263 to 269:*

*“(…) To date, the antibody dynamics in relation to active typhoid fever disease and carriage is yet to be characterized. However, both HlyE and CdtB were shown to be promising S. Typhi antigens for serological investigations (ref). Anti-HlyE IgM, IgA and IgG responses were clearly elevated in confirmed typhoid fever cases compared to healthy controls (ref). Likewise, anti-CdtB IgM responses were higher in typhoid fever cases compared to controls using recombinant CdtB in an indirect ELISA (ref). As the prime target for vaccine development, the Vi polysaccharide is the S. Typhi antigen most commonly used in serological studies (ref).”*

Minor point:

- In the “Study population” subsection of the Methods, the author should mention that the populations included in the study have not received the Vi polysaccharide-based vaccine.

*Thank you for your comments. We added the following text:*

*Lines 116 to 117:*

*“(…) Typhoid fever vaccination is not part of the national immunization schedule in the Lao PDR and it is unlikely that participants of these studies have received typhoid fever vaccination.”*

- The authors may comment if both cohorts (adult and children) had similar living

conditions (access to running water, etc).

*Unfortunately, data regarding the living conditions of the study participants were not collected during the studies. Therefore, we cannot comment on it.*

Reviewer #3:

I realize that these blood samples were from other studies, but is there any way to determine if any samples were from people vaccinated for typhoid fever? If not, could this be stated in the manuscript.

*Thank you for your comment. Please see our comment above. We included the following information in the text:*

*Lines 116 to 117: “(…) Typhoid fever vaccination is not part of the national immunization schedule in the Lao PDR and it is therefore unlikely that participants of these studies have received typhoid fever vaccination.”*

The adult blood donor age range from 17 – 40 years seems a little bit narrow. How were the individuals randomly selected? What was the overall range of the 5,018 samples that the 640 adult samples were randomly selected from for the study?

*The total number of 5018 participants was stratified according to age, sex and province. If possible, 10 female and 10 male participants were randomly selected in each age group in each province. Since there were substantially fewer numbers of participants in age groups over 40, it was decided to only include participants up to 40 years of age.*

Could more details be provided about the commercial anti-Vi IgG ELISA kit, such as were the manufacturer’s instructions followed exactly without any modifications. If modifications, what were those modifications? Wavelength? As this is a critical method of the manuscript providing the reader with enough detail to potentially replicate it is vital.

*The ELISA was conducted according to the manufacturer’s instructions. We added the following text in lines 120 to 121:*

*“To determine the concentration of anti-Vi IgG antibodies, a commercial ELISA kit (Vacczyme, Binding site, UK) was employed according to the manufacturer’s instructions. Antibody concentrations were derived from the optical density (OD) data using a standardized curve-fitting 4-parameter logistic method. (…)”*

Why was HlyE IgG ELISA included in the study? The reference cited for the in-house developed ELISA does not appear to include hlyE gene, therefore what is the specificity and sensitivity of this ELISA?

*Anti-HlyE IgG and anti-CdtB IgG ELISAs were chosen because they have been previously identified as potential biomarkers to distinguish between typhoid fever cases and healthy controls (see lines 71 to 73 in the Introduction).*

*We also included the following text in our Discussion:*

*Line 263 to 269:*

*“(…) To date, the antibody dynamics in relation to active typhoid fever disease and carriage is yet to be characterized. However, both HlyE and CdtB were shown to be promising S. Typhi antigens for serological investigations (ref). Anti-HlyE IgM, IgA and IgG responses were clearly elevated in confirmed typhoid fever cases compared to healthy controls (ref). Likewise, anti-CdtB IgM responses were higher in typhoid fever cases compared to controls using recombinant CdtB in an indirect ELISA (ref). As the prime target for vaccine development, the Vi polysaccharide is the S. Typhi antigen most commonly used in serological studies (ref).”*

Reviewer #1: Analysis as presented is well thought out and clearly done including Tables and Figures.

Major concerns:

Page 8, Line 207: We observed an upward trend of antibody titers with age in some

provinces; however, this trend t was not consistent and differed by province. Lastly, the anti-Vi IgG data was fitted as a function of age, which showed a positive relationship using Akritas–Theil–Sen non-parametric regression to account for the censored data (likelihood r =0.33, p<0.0001, Kendall`s tau = 0.21; p<0.0001) (Figure 3); suggesting that anti-Vi IgG increases with age.

Comment: what is the context of these titres in relation to active disease and carriage in endemic settings? Granted that they provide guidance on exposure, they are a good indicator and guide for deployment of vaccines to reduce carriage and transmission in the community. Can they be used for rapid detection of disease?

*Serological approaches for the diagnosis of typhoid fever have many advantages. The three S. Typhi markers used in this study were shown in other studies to be promising targets for the detection of typhoid fever (as indicated in lines 71 to 73 in the manuscript). However, the interpretation of antibody titers in relation to active disease and carriage remains challenging and none of the markers used in this study has been used in a commercial rapid test yet.*

Page 9, Line 232: The distribution of anti-HlyE IgG titers differed between Khammuane (median anti-HlyE IgG = 255.9 EU) and Phongsaly (median anti-HlyE IgG = 144.0 EU),Attapeu (median anti-HlyE IgG = 142.3 EU) and Xayabouli (median anti-HlyE IgG = 157.8 EU), and between Vientiane (median anti-HlyE IgG titer = 272.0 EU) and Attapeu and Xayabouli (Figure 4A).The median anti-CdtB IgG titres differed significantly between Khammouane (median anti-CdtB IgG= 15.3 EU) and Xayabouli (median anti-CdtB IgG = 9 EU) and between Vientiane (median anti-CdtB IgG = 16.1 EU) and Xayabouli (Figure 4B). The only significant difference in median anti-Vi IgG titers was identified between Khammouane (median anti-Vi IgG = 20.3 U/m) and Luang Prabang (median anti-Vi IgG = 7.8 U/ml) (Figure 4C).

Comment: what is the reason for the variation according to geographic distribution?

Any specific risk attributes unique to the specific study sites?

*The available data regarding the adult sample cohort is limited and only includes age, province, profession and sex. We do not have any information on risk factors or other participant characteristics. Further studies are needed to characterize the geographic variations in typhoid fever epidemiology. Any distribution would be speculation*

Reviewer #2: In general, the results are presented in a very clear and exhaustive manner.

Minor point:

- Line 207-208 (referring to Figures S3-S4): the authors wrote “We observed an upward trend of antibody titers with age in some provinces; however, this trend t was not consistent and differed by province.“

This statement should be detailed/refined. For instance, the trend seems consistent between provinces for anti-HyE IgG but not for anti-CdtB IgG. Was the population size of some provinces limiting the analysis? If yes, this should be mentioned in

the text.

*We changed the text as follows:*

*Lines 215 to 218:*

*“(…) Figure S3 and S4 show the GAM for anti-HlyE IgG and anti-CdtB IgG antibody titers as a function of age, by province in adults. We observed an upward trend of anti-HlyE IgG antibody titers with age in ~~some~~ most provinces~~;~~. For the anti-CdtB IgG data however, this trend ~~t~~ was not consistent and differed by province. (…)”*

*Lines 305 to 306:*

*“(…) Serum samples from children were only available from two provinces in the Lao PDR. In Pongsaly province, fewer serum samples were available compared to the other locations, limiting our data analyses for this province. (…)*

Reviewer #3: 5. Lines 213-225, it would be good to put in the p-Values for those significant differences.

*We added the information in lines 224 to 250:*

*“(…) There was no significant difference for any of the anti-S. Typhi antibodies between male and female children or adults. We next investigated differences in anti-S. Typhi serum IgG antibodies according to occupation and study site in adults. A Kruskal Wallis test revealed a significant difference of anti-HlyE IgG among occupation groups (p<0.0001) (Figure S5). A post-hoc Dunn´s test with Bonferroni correction determined significant differences between the general population with unspecified occupation (“other”) (median anti-HlyE IgG = 312.4 EU) and students (median anti-HlyE IgG = 157.6 EU; p<0.001), between office workers (median anti-HlyE IgG = 259.2 EU) and students (p<0.0001), and between office workers and soldiers (median anti-HlyE IgG = 187.5 EU; p<0.05). Likewise, there was a significant difference between anti-CdtB IgG titers in students (median anti-CdtB IgG = 10.1 EU) and soldiers (median anti-CdtB IgG = 13.2 EU; p<0.05), the general population with unspecified occupation (median anti-CdtB IgG = 17.1 EU) and students (p<0.01) and between students and office workers (median anti-CdtB IgG = 15.2 EU; p<0.001). There was no significant difference in anti-Vi IgG titers between the occupation groups.*

*Further Kruskal Wallis tests revealed significant differences in anti-HlyE IgG titers, anti-CdtB IgG titers, and anti-Vi IgG titers between the different provinces (p<0.0001, p<0.01 and p<0.05 respectively) (Figure 4). A post-hoc Dunn´s test with Bonferroni correction identified a significant difference between several provinces. The distribution of anti-HlyE IgG titers differed between Khammuane (median anti-HlyE IgG = 255.9 EU) and Phongsaly (median anti-HlyE IgG = 144.0 EU; p<0.05), Attapeu (median anti-HlyE IgG = 142.3 EU; p<0.01) and Xayabouli (median anti-HlyE IgG = 157.8 EU; p<0.01), and between Vientiane (median anti-HlyE IgG titer = 272.0 EU; p<0.01) and Attapeu and Xayabouli (p<0.05) (Figure 4A). The median anti-CdtB IgG titres differed significantly between Khammouane (median anti-CdtB IgG = 15.3 EU) and Xayabouli (median anti-CdtB IgG = 9 EU; p<0.05) and between Vientiane (median anti-CdtB IgG = 16.1 EU) and Xayabouli (p<0.01) (Figure 4B). The only significant difference in median anti-Vi IgG titers was identified between Khammouane (median anti-Vi IgG = 20.3 U/ml) and Luang Prabang (median anti-Vi IgG = 7.8 U/ml; p<0.05) (Figure 4C).*

Reviewer #1: Page 10, Line 252: We found that half of the participants had anti-Vi IgG antibody concentrations <7.4 U/ml, with an estimated median titer of 7.5 U/ml.

Comment: How do these titres correlate with actual typhoid disease?

*The anti-Vi titre data was interpreted according to the instructions of the commercial ELISA kit. The lower limit of detection in the anti-Vi IgG kit is 7.4 U/ml. Measurements below that threshold were considered left-censored (see lines 141 to 146). Information on how the titre data correlates with typhoid disease is not available.*

Page 11, Line 276: A follow up study specifically focusing on febrile disease and comparing blood culture data with serological testing in children in multiple provinces would allow a more accurate confirmation of the typhoid burden in children in Lao PDR.

Comment: This correlation of active disease and serology is crucial to provide a diagnostic basis for use of serological testing suggested for diagnostic purposes.

At the moment, the investigators should cite lack of this correlation as a major limitation in interpretation of their - one cannot tell if the raised levels of antigens is marker for low-grade exposure, active disease or passive carriage

*We changed the text as follows:*

*Lines 306 to 308:*

*“(…) Additionally, the correlation between typhoid fever diseases and the antibody titer data was not yet established, complicating the interpretation of the titer data considerably. W~~w~~e currently also have no cut-off for seroconversion with these antigens, so we used precise titers as a direct comparison between age groups and locations.”*

*Lines 263 to 264:*

*“To date, the interpretation of antibody titers in relation to active disease and carriage is yet to be characterized. (…)”*

Reviewer #2: While the data obtained are exhaustively analyzed and described, the

interpretation of the results remains superficial. The study – in particular, the discussion section - would highly beneficiate from a more in-depth biological contextualization and a consideration of the results in a broader context.

Here are several elements that may be further discussed:

(1) Differences observed between antibodies:

- The 3 different IgG should be further introduced. E.g., CdtB stands for Cytolethal

Distending Toxin B, and HlyE stands for Haemolysin E. Both are bacteria toxins produced by several bacteria species. The specificity of these antigens to S. Typhi should be introduced (see also “methods” comments).

*We refer to the antibodies/antigens and their characteristics in the Introduction (lines 71 and following) and provide references. We also added the following information to the manuscript:*

*Line 71 to 77:*

*“Antibodies against hemolysin E (anti-HlyE) and cytolethal distending toxin subunit B homolog (anti-CdtB) have been identified as potential biomarkers for identifying typhoid fever cases/exposure (ref). HlyE and CdtB are expressed in S. Typhi and S. Paratyphi A, but are rarely found in other Salmonella spp(ref). Additionally, antibody responses against the capsular polysaccharide Vi antigen (anti-Vi), the major component of TCVs, have also been used to assess exposure (ref). The Vi antigen is only present in S. Typhi, S. Dublin and S. Paratyphi C, but is absent from S. Paratyphi A and most of non-typhoidal serovars (ref).”*

*Indeed, we cannot exclude the possibility of cross-reactivity in our assays. Therefore, we included the following text in lines 309 to 311:*

*“Lastly, we cannot exclude the possibility of cross-reactive antibody responses, as HlyE and CdtB may be found in other bacteria strains as well (ref).”*

- A key result of the study is the absence of correlation between the anti-Vi polysaccharide IgG titers and the anti-toxin (CdtB /HlyE) IgG titers, and their different distribution in the age-stratified population. This result was unexpected and the authors should provide some hypotheses in the discussion. Could this be explained by different duration of the seroconversions? By the specificity or detectability of the antibodies? (see also “methods”comments).

*We changed the text as follows:*

*Line 282 to 286:*

*“(…)The anti-HlyE IgG and anti-CdtB IgG titres correlated reasonably well with each other in the adult and children cohort. Notably, the anti-Vi IgG data, however, did not correlate well with either of the other two antibody profiles. The low correlation between the anti-Vi IgG titers and anti-HlyE/anti-CdtB IgG titers was surprising and could be due to different durability profiles.”*

- As one purpose of the study is to orientate future survey strategies and ease the introduction of a vaccination program, the authors may provide more direct guidance on which antibody detection should be favored for future serological surveys, and possibly comment on the availability of the different antibodies and their production cost.

*Clearly these assays need more development and we think that a combination of different antigens will be the most useful. The protein antigens may be suitable for assessing background exposure and anti-Vi prevalence for immunisation status. All antigens are cheap to produce and no antibodies are required (i.e. we detect antibody not antigen)*

(2) Difference observed between age, location, occupation:

- How could the difference of serology depending on the location and occupation be

interpreted? Could it be linked with different access to running water, food origin, etc? Could the occupation be commented on in terms of Salmonella exposure? Could the province be commented on in terms of previous outbreak locations?

*Unfortunately, there is no data on potential risk factors such as water access, food consumption etc available for both the children and adult cohorts. Typhoid fever surveillance in the Lao PDR is hampered due to limited laboratory capacities. Official and confirmed information on outbreaks is lacking.*

- As the authors checked both the serology as a function of the age and as a function of the year of birth, it would be interesting to discuss if the antibody distribution in the agestratified population may result from a different exposure depending on the year (considering the date of S. Typhi outbreaks) or from the timing of biological processes.

*As stated above, data on typhoid fever outbreaks in the Lao PDR is lacking.*

- Can these results be put in context with the situation in neighboring countries with a

high prevalence of S. Typhi?

*Vaccination campaigns in both Thailand and Vietnam led to a decline in typhoid fever incidence. However, in both Myanmar and Cambodia, studies showed a comparatively high edemicity of typhoid fever (Oo et al., 2019; Pham Thanh et al., 2016; Emary et al., 2012; Kuijpers et al., 2017). These studies are based on incidence data, often from rather localized sources which limits the ability to make assumptions regarding the whole country. These data are not directly comparable as our study used a serological approach to characterize age-related S. Typhi exposure in the Lao PDR.*

Reviewer #3: 6. The authors do not discuss why there is no correlation between anti-Vi titers and anti-CdtB or anti-HlyE titers, it seems if this is a critical issue that should be discussed by the authors. The authors discuss the differences with age, but if all of these are signs of previous typhoid fever infection, would you not expect a correlation between the major polysaccharide antigen and potential toxins.

*Please see our comment above.*

Additionally, why do you think there is an age difference in the antigens that are inducing an immune response? Many enteric pathogens produce cytolethal distending toxin (CDT) is it possible that this is due to a cross-reactivity

or response to other enteric pathogens more common in children such as Campylobacter. All of these should be discussed for the paper.

*We agree that cross-reactivity is a possibility which should be referred to in our manuscript (please see also our comment above). We included the following text in lines 309 to 311:*

*“Lastly, we cannot exclude the possibility of cross-reactive antibody responses, as HlyE and CdtB may be found in other bacteria (ref).”*

7. Is it possible that the children’s samples only coming from two provinces, which were significantly higher for anti-CdtB or anti-HlyE titers could be biasing some of the results for the different provinces? This should be addressed by the authors and how this bias was avoided.

*Indeed, the children data is only available for two locations: Vientiane and Bolikhamxay. From Vientiane, there also was data from adults, but not from Bolikhamxay. We looked at a subset containing only the data from Vientiane and found that the anti-HlyE IgG and anti-CdtB IgG data followed a similar trend with age as compared to all data (see figure 1 below).*

Figure 1 Results of generalized additive model assessing anti–S. Typhi IgG antibody prevalence in children and adults from Vientiane Province as a function of age. Non-linear smooths were fitted for age in the model for anti-HlyE IgG (A) and anti-CdtB IgG (B) data. Shaded bands represent the pointwise 95%-confidence interval.

Reviewer #2: Minor points on the text:

- In the introduction, lines 61-62, the authors wrote: “A lack of longitudinal incidence

data in many countries where typhoid is suspected to be endemic is a major barrier for the introduction of typhoid conjugate vaccines (TCVs)” I suggest that the authors add a sentence explicitly explaining why this constitutes a major barrier for TCV introduction.

*Indeed, incidence data on typhoid fever disease burden should guide considerations of TCV introduction. We changed the text as follows:*

*Line 62 to 64:*

*“A lack of longitudinal incidence data in many countries where typhoid is suspected to be endemic is a major barrier for the introduction of typhoid conjugate vaccines (TCVs) since past or current typhoid fever disease burden represents the evidence base for the development of vaccination policy (GAVI, 2019). (…)*

- Direct references to given subfigure panels (for instance “Figure 1A”) are often

missing in the text. It would help the readability of the results to specify systematically which part of the figure the text is referring to.

*We changed the text accordingly.*

- Regarding the availability of data and materials, the authors state “All data generated or analyzed during this study are included in this published article and its supplementary information files.” Yet, it is unclear how to get access to the full raw anonymized dataset of the epidemiological study. As such data could be directly used for other investigations, it would be critical to provide this information.

*Due to ethical restrictions and participant confidentiality, the dataset cannot be made publicly available. However, the data can be requested from Institut Pasteur du Laos, after obtaining permission from the National Ethics Committee for Health Research (NECHR) in the Lao PDR. The contact details will be provided.*

Minor points on the Figures:

- Statistical information should be added in Figure 3.

*We changed the figure as suggested.*

- “ns” for “non-significant” could be added in Figure 5C.

*We assume the reviewer is referring to Figure S5 in the supplement material. We decided not to indicate all comparisons which were not significant with “ns” since this would considerably convolute the figure. However, we changed the legend of the figure as follows:*

“**Figure S5.** The distribution of anti–S. Typhi serum IgG titers in adults in Lao PDR by occupation

Each dot shows the measurement of an individual sample for (A) anti-HlyE IgG, (B) anti-CdtB IgG and (C) anti-Vi IgG with an underlying boxplot. Differences between groups were assessed using Kruskal-Wallis test followed by Dunn's post-hoc test with Bonferroni correction: *p<0.05, **p<0.01, ***p<0.001, ****p<0.0001. If not specified otherwise, differences in titer data were non-significant. Participants whose occupation is not specified are grouped into “other”. The dashed line in panel C represents the censoring limit, all data points below were treated as left-censored data.”

Reviewer #3:

1. The authors switch between using “typhoid fever” and “typhoid” randomly throughout the manuscript. One term should be used consistently throughout the manuscript to avoid reader confusion.

*The wording was changed to “typhoid fever” throughout the manuscript*.

2. The reference section should have genus and species names italicized for proper

formatting.

*The reference section was amended as suggested*.

--------------------

<b>Summary and General Comments</b></br></br>

Use this section to provide overall comments, discuss strengths/weaknesses of the study, novelty, significance, general execution and scholarship. You may also include additional comments for the author, including concerns about dual publication, research ethics, or publication ethics. If requesting major revision, please articulate the new experiments that are needed.

Reviewer #1: This is a manuscript that provides data on serological surveillance for typhoid specific antigen markers that maybe useful for assessment of exposure to typhoid in disease endemic settings. What is not clear is how long these markers persist in the individuals in the community and are they specific in magnitude / persistence in acute disease compared to carriage? It would have made more sense to have some longitudinal data to assess and answer this question.

Reviewer #2: The study systematically compares the presence of 3 anti–S. Typhi IgG

antibodies in the blood of Laotian of different age, sex, location, and occupation. The

outcome of the study could be a cornerstone for the development of a vaccination strategy.

Strong points: The investigation is performed properly, the manuscript is presented clearly and the conclusions well justified. Due to the low health surveillance in Lao, the collection of this data is a tour de force and offers very high public health relevance.

Weak points: The manuscript currently lacks biological contextualization of the investigation and discussion of the results in a broader context (see more detailed comments in “conclusions”).

Reviewer #3: (No Response)

--------------------

*We again thank the reviewers for their comments and suggestions and hope our work meets the criteria for publication in PLoS NTD.*

Yours sincerely,

**Professor Stephen Baker BSc DIC PhD**
