# Supplementary material for: An age-stratified serosurvey against purified Salmonella enterica serovar Typhi antigens in the Lao People´s Democratic Republic
Source: PLoS Negl Trop Dis. 2021 Dec 13;15(12):e0010017. doi: 10.1371/journal.pntd.0010017 (PMC8700045; doi:10.1371/journal.pntd.0010017)
Supplement: Attachment — Submitted filename: Hefele_L.SBaker.review.docx [file pntd.0010017.s009.docx]

PLOS Neglected Tropical Diseases

Manuscript PNTD-D-21-00419R1

The manuscript by Hefele et al., An age stratified serosurvey against purified Salmonella enterica serovar Typhi antigens in the Lao People’s Democratic Republic, is a major study of an important tropical disease that afflicts millions of people in developing countries with severe illness.

It is important to understand the prevalence of typhoid fever in an endemic country. What population subset is most affected? What are the determining factors? Age? Economic status? (trade, profession)? Gender? Others? And, how do you quantify this?

These are important questions but answering these has been beset by many problems. The answer to these questions ideally requires the routine use of a blood culture laboratory. But gaining day to day access to a laboratory culture laboratory is not practical in many developing countries. It is in answer to these problems that the international group led by Stephen Baker has adapted serological measurements to gauge disease burden while sampling the population in different provinces in Lao People’s Democratic Republic (Lao PDR).

**Significance**

This is a major study of factors that govern the incidence (and disease burden) of typhoid fever in an endemic country (Lao PDR). The study recruited a large number of people (937). They use well characterized antigens and suggest that HlyE and CdtB might be better alternatives to the capsular polysaccharide Vi antigen to characterize to describe the disease burden in Lao PDR and might be an alternative target for vaccine development against S Typhi.

This work, despite some limitations, provides clear data that will help future public health decisions and cost effective strategies in the design of new generation vaccines to address the disease burden.

The **methodology** is well described and adequately backed by literature references. The plots neatly describe the data. This together with the large study size allows the authors to draw convincing and useful conclusions. The results clearly show, as reported, that antibody responses against typhoid fever antigens HlyE and CdtB are notably higher in children than in adults. However, when using the bacterial polysaccharide antigen Vi, antibody responses are higher in adults than in children.

**Major issues - none**

Importantly, the authors, Hefele et al. address all major issues that the prior reviewers brought up satisfactorily. This reviewer has no major issues with the manuscript. It is my hope, however, that future studies will try to collect samples to a repository for future study, especially in light of the difficulty in carrying out longitudinal studies.

Minor issues

There are “a couple” of typos that the authors might want to fix -

line 32: ‘anti-HylE’ should read ‘anti-HlyE’

line 55: ‘burden typhoid’ should read ‘burden of typhoid’

line 57: ‘anti-HylE’ should read ‘anti-HlyE’

**Recommendation**

I am impressed by this manuscript. It adds valuable new data to the published domain and it is clearly written. I recommend to the Editor that this submission be accepted and published.

**ACCEPT**
