# Supplementary material for: An age-stratified serosurvey against purified Salmonella enterica serovar Typhi antigens in the Lao People´s Democratic Republic
Source: PLoS Negl Trop Dis. 2021 Dec 13;15(12):e0010017. doi: 10.1371/journal.pntd.0010017 (PMC8700045; doi:10.1371/journal.pntd.0010017)
Supplement: Attachment — Submitted filename: Cover letter_041021.doc [file pntd.0010017.s010.doc]

Professor Stephen Baker

Sgb47@medschl.cam.ac.uk

4^th^ October 2021

Re: Submission to PLoS neglected tropical diseases

Dear editor,

On behalf of all authors, I wish the midified manuscript entitled **“An age-stratified serosurvey against purified *Salmonella enterica* serovar Typhi antigens in the Lao People´s Democratic Republic”** by Lisa Hefele et al, for which I am the corresponding author, to be considered for publication as a research article in PLoS neglected tropical diseases. We thank the editor and reviewers for their support and have addressed each comment in turn below.

Reviewer #2: The authors have replied to all the points previously raised in their point-by-point answer. However, in most cases, they could not directly improve the results and conclusions within the manuscript, but rather detailed in their rebuttal the current limitations that restricted the study (lack of current knowledge on the antibody responses, lack of epidemiological information in Lao, etc.). Given these circumstances, the authors pushed the analysis and interpretation to their best extend and avoided drawing conclusions or interpretations that could not be directly supported by the data.

Reviewer #3: Conclusions from the study are supported by the data from the study, but a little more in depth thought as to the meaning of the results would help to strengthen the manuscript.

Reviewer #4: Conclusions are borne out by the data. Limitations of the study are also addressed by the authors.

*We thank the reviewer for their comments. We expanded our Discussion to address the suggestions and issues raised by the reviewers (see our responses below).*

**Summary and General Comments**

Use this section to provide overall comments, discuss strengths/weaknesses of the study, novelty, significance, general execution and scholarship. You may also include additional comments for the author, including concerns about dual publication, research ethics, or publication ethics. If requesting major revision, please articulate the new experiments that are needed.

Reviewer #2: While the limited conclusions of the study may impair its visibility, it also highlights the necessity to support and develop similar studies until reaching a broader understanding of typhoid epidemiology. However, I regret that the authors were unable to consequently revised the text to contextualize their study as suggested. This would have made the manuscript more attractive for a broader audience (only minor edits were implemented in this regard despite the editor's recommendation to significantly revise the manuscript). In its current state, as the manuscript mainly displays epidemiological observations but no or limited interpretations of the results, it may fit best a journal dedicated to epidemiological reports.

*We now include subset analyses from Vientiane, for which samples from both children and adults were available and revised the text in our Discussion to further comment on antibody dynamics (see our response below).*

Reviewer #3: Overall, I think the manuscript has been greatly improved, but there are still a couple of minor issues that should be resolved prior to publication:

As the central finding of the study is that children have higher titers for HlyE and CdtB compared to adults, however this includes adults from numerous provinces and children from only two provinces. It would be interesting to see if this continues to be true by narrowing the analysis down to just the two provinces that all the children samples came from for the study (e.g. Vientiane & Bolikhamxay).

*In our first reply to the issues the reviewers raised, we included a graph showing the generalized additive models for anti-HlyE IgG and anti-CdtB IgG data by age in the subset for Vientiane which includes samples from both children and adults. In the Vientiane subset, the antibody titers follow a similar trend when compared to the whole dataset.*

*We now included our subset analyses in the supplement of our manuscript and made the following changes to the text of the manuscript:*

*Lines 190 to 192:*

*“Data from both children and adults was available from Vientiane. The same difference between the distributions of antibody titers between children and adults were observed in this subset (Figure S2).”*

*Lines 224 to 227:*

*“In addition, we assessed the relationship between antibody titers and age in the subset for Vientiane for which data from both children and adults was available. A similar pattern was observed: Anti-HlyE IgG and anti-CdtB IgG titers were highest in children and decreased with age, while anti-Vi IgG titers were highest in adults (Figure S6).”*

*Additional graphs for the supplement section:*

**Figure S2**. The distribution of anti–S. Typhi serum IgG titers in children and adults in Vientiane, Lao PDR

Each dot shows the antibody titer of an individual sample for (A) anti-HlyE IgG, (B) anti-CdtB IgG, and (C) anti-Vi IgG with an underlying boxplot. The dashed line in panel C represents the censoring limit, all data points below were treated as left-censored data. Differences between groups were assessed using Wilcoxon rank sum test followed by Dunn's post-hoc test with Bonferroni correction: ***p<0.001, ****p<0.0001.

**Figure S6.** Results of generalized additive and linear models assessing anti–S. Typhi IgG antibody prevalence in children and adults in Vientiane, Lao PDR as a function of age

Non-linear smooths were fitted for age in the model for anti-HlyE IgG (A) and anti-CdtB IgG (B) data. The tick marks on the x-axis are observed data points. In panel C, the Akritas-Thiel-Sen regression line relating to the anti-Vi IgG titer data as function of age was plotted in order to account for the censored values (censored observations were plotted as vertical dashed lines). %dev. = the percent of the total model deviance explained.

I would like to see them expand the thoughts on the low correlation between anti-Vi titers and anti-HlyE/anti-CdtB titers, as this seems critical to the study, but the authors do not offer a very detailed explanation other than different durability profiles. Is there anything in the literature to support this thought? Anything in the literature to offer alternative ideas?

*As we indicated in our previous reply to the issues raised by the reviewers, data showing IgG antibody dynamics in response to typhoid fever disease and carriage is extremely limited. We could not find any prior publication detailing the correlation of these IgG antibodies with each other.*

*One publication reports the correlation of IgM measurements between anti-CdtB IgM and anti-Vi IgM and we included the reference in our discussion section.*

*Lines 292 to 295:*

*“In contrast to the low correlation (tau -0.09; all data) between anti-CdtB IgG and anti-Vi IgG in our study, the IgM measurements against both of these antigens showed a moderate correlation (rho = 0.77) [7]. Data regarding the relationship between anti-S. Typhi antibody responses, in particular IgG responses, is currently lacking, indicating the need for further investigations.”*

*Regarding the relationship between antibody titers and age:*

*In our discussion (lines 279 to 280), we refer to other reports which also show a positive association between anti-Vi IgG titer and increasing age.*

*However, we could not find any other publication on the relationship between anti-HlyE IgG and anti-CdtB IgG titer and age, emphasizing the importance of further studies investigating S. Typhi antibody dynamics.*

For the difference in children versus adults that is opposite from the hospital based study in Vientiane, what do other typhoid fever studies in other countries suggest about the results? Have other studies found differences between serum based versus hospital based studies in other countries? Overall, I think a little bit more thought on the meaning of the results is needed for the discussion prior to publication. Otherwise it is a solid paper.

*While there are hospital-based studies in other counties, serum studies are rare given the difficult interpretation of antibody responses. Our anti-HlyE and anti-CdtB IgG data suggests higher exposure in children compared to adults, which contrasts hospital-based incidence studies from the Lao PDR. However, globally, the incidence of typhoid and paratyphoid fever is highest in children, peaking between 5 and 9 years (Stanaway et al., 2019). Typhoid fever incidence by age varies between high and low incidence settings. We included this information in our discussion and changed the text as follows:*

*Lines 304 to 308:*

*“Age-related Typhoid fever incidence patterns vary between high and low incidence settings: the incidence is typically highest in children in high incidence settings but more equally distributed in low incidence settings. In the global context, children are disproportionally affected by typhoid and paratyphoid fever with the highest incidence occurring in children between 5 and 9 years [ref].”*

--------------------

*We again thank the reviewers for their comments and suggestions and hope our work meets the criteria for publication in PLoS NTD.*

Yours sincerely,

**Professor Stephen Baker BSc DIC PhD**
