# Supplementary material for: An age-stratified serosurvey against purified Salmonella enterica serovar Typhi antigens in the Lao People´s Democratic Republic
Source: PLoS Negl Trop Dis. 2021 Dec 13;15(12):e0010017. doi: 10.1371/journal.pntd.0010017 (PMC8700045; doi:10.1371/journal.pntd.0010017)
Supplement: Attachment — Submitted filename: Hefele_L.SBaker.review_2.docx [file pntd.0010017.s011.docx]

PLOS Neglected Tropical Diseases

Manuscript **PNTD-D-21-00419R2**

**An age-stratified serosurvey against purified *Salmonella enterica* serovar Typhi antigens in the Lao People’s Democratic Republic**

The manuscript by Hefele et al. address the prevalence of typhoid fever in the Lao People’s Democratic Republic (Lao PDR). This is an important study. Despite some limitations and limited scope, the data is valuable and defines future research questions. It is expected that the study will also help in making future public health decisions.

The authors have addressed my questions and those raised by the other reviewers.

**Significance**

This is a major study even if limited scope.

**Major issues**

None

The authors address all of the questions raised by the reviewers. It remains my hope that in this and future studies there are plans to build a repository of samples.

**Minor issues**

None

**Recommendation**

I recommend to the Editor that this revised version be accepted.
